# Rethinking Health Recommender Systems for Active Aging: An Autonomy-Based Ethical Analysis

**DOI:** 10.1007/s11948-024-00479-z

**Published:** 2024-05-27

**Authors:** Simona Tiribelli, Davide Calvaresi

**Affiliations:** 1 Department of Political Sciences, Communication, and International Relations, University of Macerata, 62100 Macerata, Italy; 2 Institute for Technology and Global Health, PathCheck Foundation, 955 Massachusetts Ave, Cambridge, MA 02139 USA; 3 University of Applied Sciences and Arts Western Switzerland (HES-SO), Rue de l′Industrie 23, 1950 Sion, Switzerland

**Keywords:** Health recommender systems, AI ethics, Autonomy, Active aging, Persuasion technology

## Abstract

Health Recommender Systems are promising Articial-Intelligence-based tools endowing healthy lifestyles and therapy adherence in healthcare and medicine. Among the most supported areas, it is worth mentioning active aging. However, current HRS supporting AA raise ethical challenges that still need to be properly formalized and explored. This study proposes to rethink HRS for AA through an autonomy-based ethical analysis. In particular, a brief overview of the HRS’ technical aspects allows us to shed light on the ethical risks and challenges they might raise on individuals’ well-being as they age. Moreover, the study proposes a categorization, understanding, and possible preventive/mitigation actions for the elicited risks and challenges through rethinking the AI ethics core principle of autonomy. Finally, elaborating on autonomy-related ethical theories, the paper proposes an autonomy-based ethical framework and how it can foster the development of autonomy-enabling HRS for AA.

## Introduction

Advances in digital technology and Artificial Intelligence (AI) are fostering the evolution of digital healthcare systems. In this context, continuous and ubiquitous support is enhancing personalization and preventive patient-centered care (Calvaresi et al., 2017a). The users of such systems are supposed to progressively become more autonomous, adopt healthy behaviors, and maintain themselves active and healthier over time (De Croon et al., 2021). Such processes can entail a user’s behavioral change (Orji & Moffatt, 2018). To support it, health recommender systems (HRS), often leveraging persuasion technologies (PT)—i.e., rule-based behavioral strategies –, are increasingly considered the most promising approaches (De Croon et al., 2021; Hunter, 2018). In particular, active aging (AA) is a domain in which PT-enabled HRS can play a crucial role. Indeed, aging individuals (approaching frailty) will inevitably face growing health-related matters as they deal with more complex needs (e.g., chronic conditions management), metabolic changes (involving dietary adaptions), reduced resources/information accessibility, possible cognitive degradation, etc. Therefore, assistive technologies (including HRS) target aging individuals as main users/service-consumers (Calvaresi et al., 2017a; Torres et al., 2023; De Croon et al., 2021; Oliva-Felipe et al., 2018). However, to date, there is no unique and reconciling definition of AA. Nevertheless, its multi-faceted nature is generally agreed upon. Indeed, HRS are widely acknowledged as supporting the several dimensions of AA (Fernández-Ballesteros et al., 2013). The underlying contributions span over counteracting loneliness (Holwerda et al., 2012), promoting social support (Tomaka et al., 2006), enhancing social/family relationships (Victor et al., 2000), facilitating social activity and fostering a sense of personal fulfillment (Creecy et al., 1985), strengthening healthy habits (McPhee et al., 2004)—such as smoking cessation and better nutrition (Pirlich & Lochs, 2001), including healthy active motor-habits (Cvecka et al., 2015), stimulating cognitive activities (Tardif & Simard, 2011), and treating/managing diseases and pathologies (e.g., diabetes and cancer) (Longo et al., 2010; Manzo et al., 2021). Indeed, AA goes beyond the health dimension of well-being in aging. It concerns individual (e.g., well-being derived from living a meaningful life), socio-relational perspectives (e.g., well-being related to participating in social life and being in meaningful relationships), and security (feeling safe) (Fernández-Ballesteros et al., 2013; WHO, 2023).

Elaborating on AA’s key elements and dimensions outlined above from an HRS design perspective, we claim that HRS *should* contribute to four aspects (i.e., physical, personal, social, and cognitive) to foster AA. With respect to such aspects, HRS contribute as follows.

*Physical* (PH)—provide knowledge-based health-related motor stimuli and recommendations tailored to the user’s needs and capabilities while preventing human deskilling; *personal* (PE)— ensure a fair set of meaningful choices and options that do not constrain or erode user’s freedom of choice; *social* (SO)—promote interactions among individuals in similar conditions to foster motivation, support, and empathy, preventing self-isolation and social pressure; and *cognitive* (CO)—provide user-friendly, intuitive, and pervasive interfaces and interactions that do not understimulate nor overwhelm the user perception, elaboration, and actuation capabilities (most relevant causes for early tech abandon).

The effectiveness and efficiency of current HRS can also come at the expense of the users’ autonomy (see Sect. "Rethinking HRS Challenges Based on the Ethics Principle of Autonomy"). Indeed, although HRS can empower users’ autonomy in AA, especially in terms of self-control and independence, they can also raise ethical risks and concerns that can undermine AA. An example can be the unfair reduction of meaningful opportunities due to biased correlations and socio-relational exposure, possibly leading to alienation/isolation (Pariser, 2011; Knees et al., 2024; Valentine et al., 2023). Such risks can only be understood through a more complex *multidimensional* concept of autonomy  (Prunkl, 2022; Tiribelli et al., 2023b). This paper (i) investigates HRS and proposes (ii) an understanding of the AI ethics principle of autonomy to elicit and circumvent the threats affecting AA, and (iii) possible mitigation strategies.

The remainder of the paper is organized as follows. Section "Health Recommender Systems: Technical Aspects and Ethical Challenges" briefly introduces HRS’ main technical features and their role in health behavioral change in AA. In turn, it highlights potential ethical risks that HRS can generate for individuals pursuing health-related goals and contextualizes them into the AA’s key aspects. Section "Rethinking HRS Challenges Based on the Ethics Principle of Autonomy" draws on ethical theory to show how an adequate understanding of the AI ethics principle of autonomy helps to categorize, prevent, and/or mitigate such ethical risks and design HRS for AA. Section "From Theory to Practice: Enacting Autonomy in Next-Gen HRS" shows how to rethink the next-generation HRS via the ethical principle of autonomy and how the latter helps to consider a series of strategies to counteract HRS-related autonomy threats. Finally, Sect. "Conclusions" concludes the paper.

## Health Recommender Systems: Technical Aspects and Ethical Challenges

According to De Croon et al. (2021), recommender systems (RS) are software tools and techniques providing suggestions for items of interest for a given user. We experience RS daily, receiving suggestions affecting several decision-making processes, such as items to buy, music to listen to, and news to read. HRS are a specialization of RS. As they operate in the health context, HRS recommend items of interest that generally consist of non-confidential, scientifically proven, or at least generally accepted health-related and/or medical information.

Conventional RS have boosted, among others, marketing, e-commerce, and overall consumerist behaviors (Calvaresi et al., 2022). However, ingrained in modern life, commercial RS have remarkably contributed to the development of unhealthy habits (i.e., excessive consumption of energy/sugar-dense food and alcohol and use of unhealthy sets of recipes) that can last over time (Trattner & Elsweiler, 2017; Calvaresi et al., 2022). HRS should fight such tendencies by promoting prevention, awareness, and overall healthy behaviors (De Croon et al., 2021). Indeed, HRS mainly operate in lifestyle, nutrition, general health, and condition-specific application scenarios (De Croon et al., 2021). Overall, HRS span from wellness and well-being to medical application scenarios. Clearly, the implications and responsibilities vary remarkably in such a broad spectrum. Nevertheless, they can all be concerned when focusing on HRS applications in AA. For instance, there are HRS designed to enable AA by counteracting elderly social isolation (e.g., by providing multi-modal coaching for social activities  (Dimitrov et al., 2019)), improving nutrition habits (e.g., providing tracking functionalities and “guiding” recommendations to observe a good macro-nutrients balance) (Espín et al., 2016; Calvaresi et al., 2021b), promoting psycho-physical activity (e.g., proposing general-purpose and personalized physical tasks (Nassabi et al., 2014)), including the cognitive activity (e.g., for equilibrium maintenance: HRS that provide reminders and personalized exercises to stimulate muscles and cognition skills  (Calvaresi et al., 2021)), and allowing tele-rehabilitation (e.g., HRS providing monitoring and guidance to recover a good range of motion, such as in the case of older individuals who have undergone lower-limbs surgery) (Buonocunto et al., 2018). Finally, several solutions have tried to provide longitudinal (multi-dimension) contributions, employing argumentation and rewarding mechanisms (Herpich et al., 2017), fighting addictions (e.g., smoking cessation) from both a social and physical health perspectives - by providing support in the monitoring and the craving phase (Calvaresi et al., 2019), and via automated (AI-based) and manual (formulated by doctors) recommendations and guidelines (e.g., for cancer survivors (Manzo et al., 2021)).

From a technological perspective, RS and HRS partially share some core elements. For example, both RS and HRS can rely on symbolic-based reasoners (i.e., rule-based state machines), data-driven engines (i.e., machine learning/deep learning—ML/DL models), and hybrid infrastructures leveraging both the approaches (i.e., via neuro-symbolic integration (Spillo et al., 2022)).

Moreover, RS can be non-personalized (i.e., not requiring any prior knowledge about any specific user (Falk, 2019) or personalized (i.e., requiring a remarkable amount of knowledge about the targeted user). Instead, HRS are intended to be strongly personalized due to profile- and context-crucial information. Non-personalized RS leverage generic information (i.e., items’ popularity, novelty, price, and distance) to sort the possible recommendations. For example, basket modeling & analysis is used in retail to identify complementarity (i.e., items to recommend as usually bought together) (Yıldırım et al., 2020; Lin et al., 2002). Personalized (H)RS leverage users’ details, including demographics, location (i.e., geofencing), language, preferences, and possibly aggregated behaviors to have a deeper understanding of the user (Schäfer et al., 2017; Logesh et al., 2019). Such data are usually obtained by tracking and profiling the users (Wang et al., 2021; Iwata et al., 2007). Here, *rating* is a popular mechanism to determine users’ preferences and qualify an item explicitly or implicitly (if the preference is deduced from the user’s behavior) (Guo et al., 2014; Oard et al., 1998). Moreover, as summarized in Calvaresi et al. (2021b), the most adopted data-driven techniques in HRS are: *collaborative filtering*(CF), which consists of leveraging users’ similarities and ratings (Jannach et al., 2010); *content-based filtering*(CB), which consists of recommending similar items based on similar profiles’ previously liked items Sánchez Bocanegra, Sánchez Laguna, and Sevillano (2015); *knowledge-based recommendation*(KB), where the recommendation is based on the user preferences and constraints (Felfernig & Burke, 2008), and *hybrid recommendation* (HR), which combines the techniques mentioned above (Ricci et al., 2011)). In short:

CF: Uses the collected data to identify similarities between items and user profiles and, in turn, computes the expected ratings of unseen items (Aggarwal et al., 2016; Chen et al., 2018). CF algorithms can be classified as *memory-based* (Karabadji et al., 2018), *model-based* (matrix factorization, tensor completion) (Luo et al., 2014), *hybrid CF* (combining model-based and memory-based) (Zhang et al., 2017), and *deep learning-based* (Wei et al., 2017) approaches. For example, Sahoo et al. (2019) utilized CF to correlate patients (i.e., to propose physical exercises) based on their personal interests, feedback on therapies, etc.).
CB: Uses the items’ underlying characteristics to provide new recommendations—mostly suitable if the user is directly interested in them (Wang et al., 2018). For example, CB-based HRS are used to support health consumers looking for multiple sources when searching for health information online (Longo et al., 2010).
KB: Conceals the knowledge about a given domain—mostly suitable if variability and personalization are broad (requiring both domain and item-specific knowledge) (Tarus et al., 2018). For example, it is employed by systems monitoring emotional health to detect users with potential psychological disturbances (i.e., depression and stress) and to send happy, calm, relaxing, or motivational messages contextually (Rosa et al., 2018).
HR: Combines two or more approaches. For example, in a CF approach, the dependency on ratings entails disfavoring unrated items. Nevertheless, combining CF and KB approaches (if the items contain attributes) could solve the limitation (Dong et al., 2017). For example, it is employed by applications (i.e., smartphone-based virtual assistants) that process a broad range of vital/wellness parameters to understand if contacting a doctor or performing further analysis is recommended (Jamshidi et al., 2018).

Overall, although hybrid approaches seem to be the most promising option from current literature in the field, to date, in practice, RS mainly rely on data-driven approaches, and HRS mainly rely on rule-based finite state machines—mainly given the complexity and sensitivity of the input data and effects on the users.

Such a tendency is particularly motivated by the presence of PT within the HRS reasoning engine. It is worth recalling that persuasion is intended as “an activity that involves one party trying to induce another party to believe or to do something" (Hunter, 2018). In HRS, the persuasion usually targets abandoning unhealthy behaviors in favor of what is suggested (what is considered healthy and a good solution for the given user condition). Persuasion strategies are mostly formulated as symbolic connections (i.e., state machines) that ensure more control of the process. Moreover, it is necessary to distinguish PT from other forms of influence on freedom of choice, which cannot be considered legitimate. Unlike manipulation or coercion, persuasion affects the architecture of choices by providing pre-known (possibly extended) alternatives without undermining freedom of choice (Carli et al., 2022).

While such techniques have been shown to be particularly functional in triggering a behavioral change in health and for HRS’ tasks, as outlined  (Calvaresi et al., 2022), such techniques can also raise a number of ethical risks and challenges that, if untackled, can undermine individuals’ well-being—in particular in aging conditions. The ethical risks underlying such challenges indeed become increasingly relevant with respect to those raised by conventional RS (Milano et al., 2020), considering their high-sensitive application field.

As follows, we highlight some of the major ethical challenges that HRS, as increasingly prototyped as HR in current literature (De Croon et al., 2021), ought to address to ensure their ethical development (i.e., their design and implementation to ensure people’s well-being). Then, we show how such ethical challenges (EC), if unaddressed, can undermine AA’s key dimensions previously outlined. HRS should:

EC1: Avoid inaccurate recommendations. HRS must be benevolent. This means that HRS target users’ health and therefore provide suggestions that must not harm the users’ health. An example can be recommending too complex or advanced physical exercises, just leveraging user self-reporting inputs, insofar as they can lead to serious injuries. Benevolence includes accuracy with respect to individuals’ core beliefs and values.
EC2: Ensure privacy: the generation of personalized health recommendations entails access to (sensitive) personal information. As mentioned above, CF has been shown to be vulnerable to data leakage in the inference phase (Calandrino et al., 2011; Ji et al., 2020). HRS are strongly characterized by a trade-off between recommendations’ accuracy and how much the users are willing to share about themselves. The current research direction is a layered notion of privacy per user group (Xin & Jaakkola, 2014). For example, systems leveraging third-party services might run into data leakage, exposing data to partially authorized personnel (i.e., within a given HRS, a doctor must only be allowed to visualize the info of patients who gave them specific consent) or using personal data as not initially declared/intended.
EC3: Safeguard personal identity and freedom of choice: HRS might (a) intentionally restrict users’ freedom of choice with biased recommendations and/or a reduced (sub)set of options and (b) reshape the users’ community, leading to echo chambers and filter bubbles—a risk to which are exposed HRS providing social-integration/support features (Kampik et al., 2018). Examples can be (i) the over-simplifying of the food items list to be recommended, (ii) vehiculating the community’s interest towards third-party products/services pushing for mere consumeristic interests, and (iv) proposing a recommendation (medically correct) that might counter the user’s moral/cultural values.
EC4: Reduce the HRS opacity: current HRS engines employ ML/DL predictors “as is” and do not provide transparency on their internal mechanisms (i.e., no info about how/why a given recommendation is/has been produced). Such opacity instills mistrust and a lack of accountability (Graziani et al., 2022; Anjomshoae et al., 2019). For example, as of today, users cannot ask how/why a given recommendation (e.g., medication, food item, activity, therapy, nearby shop, or restaurant) is proposed and have no power over it.
EC5: Avoid unfairness: skewed and biased data sets and inappropriate contextual constraints can yield unfair recommendations. To mention a few, a user might receive recommendations (i) (un)intentionally committed to brands, activities, or doctors, including getting proposed over-the-counter drugs brands—possibly more expensive—over others less known/publicized—and possibly cheaper—on a regular basis), (ii) that are unattainable due the possible user’s limited access to healthcare services or information, physical disabilities, lack of internet access, or language barriers, and (iii) that suggest solutions that are insensitive of the user’s culture.
  Unfortunately, the identification, isolation, and exclusion of such biases to avoid unfair recommendations are strongly hampered by the opacity of today’s HRS and their related training mechanisms.
EC6: Avoid social pressure and socio-relational impoverishment: as already mentioned in EC3, polarization is considered one of the most dangerous side-effects of using HRS (Knijnenburg et al., 2012; Konstan & Riedl, 2012)—unfortunate (H)RS byproduct since their early adoption (Van Alstyne et al., 1996). Social networks have exacerbated (H)RS’ effects and set themselves as an (almost unique) source of news and information, dramatically boosting filter-bubbles (Bozdag & Van Den Hoven, 2015) and echo-chambers (Levy & Razin, 2019) and worsened the already existing polarization (i.e., societal). For example, aging individuals coping with smoking cessation campaigns that include social aspects conducted over social media are extremely emotionally vulnerable. Hence, they are dramatically exposed to manipulation/coercion. Moreover, HRS can end up strengthening socio-relational isolation and impoverishment. For example, promoting excessively isolating activities—possibly due to users’ mobility restrictions—can lead users to loneliness and malaise and harm AA’s social dimension.

Finally, Table 1 presents a possible mapping of AA aspects with the elicited ethical challenges. In particular,

PH: HRS might push the user beyond their capabilities and safety boundaries to reach a goal. Indeed, many HRS rely on self-reported and possibly missing data, potentially presenting a more “optimistic” picture than reality. Therefore, naive or overly mechanistic approaches can endanger the user rather than promoting their best interest. Moreover, having limited or unbalanced sets of practices (solely focusing on given tasks or entirely ignoring others) can cause the user’s progression to drift in unforeseen/unwanted directions.
PE: Recommendations might include elements asking users to violate or infringe their personal values and beliefs. This can occur if the user model disregards some elements (i.e., religion, nutritional orientation, social commitments, and societal customs) or if the user is misclassified. On the one hand, using users’ sensitive information might violate personal standing. For example, most users usually agree to share some or all of their information to improve the system or enhance medical understanding. Nevertheless, data misuse could represent a risk of fostering commercial interests at the user’s expense (extending/shifting from what they have originally agreed with). On the other hand, assuming coherency in the suggestions, the user might not perceive the rationale behind them, and current HRS fail to engage in argumentative interactions to explain the system’s recommendations and decisions. Eventually, users can come to question HRS’ fairness (i.e., hidden promotion of consumeristic interests –brands over others– via recommendation), and, to date, HRS provides no exhaustive answers or clarifications regarding their decision-making.
SO: Personal information can be used by HRS to foster users’ adherence (e.g., via group chats over social media or gamification—leveraging the social factor). However, despite the possible positive outcomes obtained in most cases, more attention must be paid to shielding specific aspects that outlier users would like to protect rather than share. Indeed, such information might be misused by individuals of the same “community/society” against their owners, inducing self-isolation rather than the hoped/expected integration.
CO: HRS can provide both physical and mental assistance. From a cognitive perspective, some tasks, procedures, and work/data flows must be more flexible and adaptable to a specific audience. For example, the language, formulation, and length of the consent forms are often more confusing than informative, leading the user to blindly accept them to be able to use the system’s functionalities. Once again, such formulations are static and generic (one-size-fits-all) and are not processed by all users in the same way.

**Table 1:** Mapping active aging aspects—HRS ethical challenges

AA aspects	HRS ECs
PH	EC1,EC3
PE	EC1, EC2, EC3, EC4, EC5, EC6
SO	EC1, EC5, EC6
CO	EC1, EC2, EC5

## Rethinking HRS Challenges Based on the Ethics Principle of Autonomy

We have outlined that HRS can be a valid technological ally supporting AA. Indeed, several studies mentioned above have shown that HRS can provide tangible support for several AA-related tasks and activities (e.g., enabling healthier habits and health-and-care self-management) (De Croon et al., 2021; Torres et al., 2023; Calvaresi et al., 2017a). However, we have also shown that HRS can raise several ethical risks and challenges. Such risk exposure can jeopardize the HRS benefits, producing effects damaging individuals’ well-being, especially during aging. Thus, it seems crucial to understand how to address the ethical challenges (EC1-to-EC6) and, therefore, prevent the aforementioned ethical risks to fully harness HRS potential in AA. Indeed, as previously outlined, AA concerns people’s well-being in aging beyond the sole health domain. Rather than considering HRS as a *panacea* solving all the issues related to aging, it is vital to adopt a balanced perspective on HRS in terms of their opportunities and downsides to understand their role in supporting AA.

Therefore, the question that arises concerns how HRS engineers can design such tools to take advantage of their value for AA while preventing the ethical risks mentioned. Also, insofar as ethics is concerned, it sounds legitimate to ask whether it is possible to rely on ethical principles, guidelines, and/or criteria to tackle the ethical risks and challenges outlined above.

In this section, we claim that HRS ethical challenges can be understood as challenges to the AI ethics principle of autonomy if the latter is adequately understood. Specifically, we show how autonomy requires diverse sub-conditions to be met, whose consideration helps to categorize such risks and challenges. Therefore, we will unpack the ethical principle of autonomy, drawing insights from ethical theory rooted in moral philosophy. By doing so, we will show how our ethical inquiry can offer an autonomy-based ethical framework to steer the design of near-future HRS that prevent and/or mitigate the above-mentioned ethical risks and challenges and eventually promote AA.

### Why is Autonomy Crucial in HRS for AA?

Autonomy plays a central role in AA. As pointed out by the World Health Organization (WHO, 2023), the maintenance and the promotion of autonomy are crucial for enabling AA. Overall, autonomy is referred to as (i) the ability to control and make personal decisions about one’s life according to one’s own rules and preferences (autonomy as control and self-determination), (ii) independence - that is, the capacity to live independently in the community, with no or little help from others  (WHO, 2023; Paúl et al., 2012), (iii) connected to the quality of life - as people age, their quality of life is determined by their ability to maintain autonomy and independence, where the quality of life also includes physical health, psychological state, personal beliefs, and social relationships (Paúl et al., 2012), and by (iv) the healthy life expectancy (how long people can expect to live autonomously), that is, without disabilities (p. 3).

Moreover, autonomy is recognized as a paramount issue in the age-tech field based on AI and algorithms (Ho, 2020; Rubeis, 2020; Rubeis et al., 2018; Sixsmith et al., 2013). From this literature, a widely embraced idea is to design self-tracking and personalized health systems by considering, on the one hand, how they can promote the autonomy of the elderly (Ho, 2020), especially in terms of engagement, self-management, and self-determination on health-related issues (Sharon, 2017; Müller et al., 2010; McLean et al., 2013; Vassilev et al., 2015; Topol, 2015), and on the other hand, how they can undermine it—by raising risks such as profit-driven health surveillance and/or social pressure, users’ informed consent bypassing, and privacy infringements (Lupton, 2013; Rubeis, 2020).

Similar ethical challenges are also at the core of the nascent debate on the ethics of RS (Milano et al., 2020; Varshney, 2020). Here, autonomy is mainly understood as human self-determination and is claimed to be the core ethical principle for the future design of ethical RS. This is because the benefits, main opportunities, and risks raised by RS are considered to be intertwined with autonomy (Varshney, 2020). Indeed, RS can empower people’s autonomy as they help users to navigate informational overload saving time and cognitive resources. This is also relevant for HRS. For instance, consider HRS used as personal health virtual assistants (Varshney, 2020) or as embedded in web-based platforms to access health information/recommendations. According to Sezgin and Özkan (2013), such tools have a significant role in filtering information for users’ self-diagnostic searches. Some contextual examples proposed in such a study are HRS used by physicians for diagnostic and educational purposes, such as HRS suggesting online health resources (HealthyHarlem) and HRS-based educational resources with patient records (MyHealthEducator). Other examples include HRS in mHealth apps connecting users with doctors or healthcare providers (HCP) using filtering options based on people’s tracked profiles. Other examples in the telemedicine field include HRS used to recommend health treatments in support of medical diagnoses, as in the case of the UK Babylon health app (Iacobucci, 2020; Tiribelli et al., 2023b). Such opportunities are even more relevant to enhancing the autonomy of aging individuals (with weakening cognitive skills). However, such users both benefit from HRS as well as might be more affected by the residual ethical risks they pose. As highlighted in studies (Milano et al., 2020; Varshney, 2020), the risks raised by RS are also strictly intertwined with autonomy (Whittlestone et al., 2019; Varshney, 2020). Such risks range from human deskilling or cognitive atrophy (independence loss), due to tasks’ over-delegation, to social homogenization and cultural diversity impoverishment. The latter happens when RS suggest similar or identical options/items for certain segments of populations and thus reducing the qualitative diversity of options and diversified social exposure that are critical for adequate and genuine decision-making (Fleder & Hosanagar, 2009; Tiribelli, 2023) — creating filtering or epistemic bubbles (Pariser, 2011).

Overall, the principle of autonomy is central to the design of RS. Indeed, to be accurate and effective, RS cannot avoid intervening in some ways on users’ autonomy to some extent (Cowls et al., 2019), such as reducing options or extracting users’ crucial information to contextualize recommendations. Such consideration is pivotal for RS used in the health industry and for health or medical purposes. As personal health information is key to developing accurate recommendations, the risks of undermining users’ autonomy increase - especially for already vulnerable groups  (Milano et al., 2020; Varshney, 2020; Mittelstadt et al., 2016). Nonetheless, people might be willing to take such risks by voluntarily choosing to use such technology and consciously ceding/renouncing some of their autonomy in exchange for other potential benefits stemming from such use.

In addition to being recognized as a key principle for AA (WHO, 2023), autonomy is one of the most acknowledged ethical principles in the field of ethics of AI and algorithms (Jobin et al., 2019) in general and as applied to health (Guidance, 2021)^1^. While the relevance of autonomy in the fields intersected by our inquiry is evident, some outline how such a core AI ethics principle still tends to be vague or undefined. In other words, it is not clear what the respect and promotion of autonomy truly demand for the ethical design of AI (Prunkl, 2022) and RS (Prunkl, 2022).

In summary, autonomy is acknowledged as a crucial ethical principle for AA, as well as for the design of AI and RS (in general) or health RS (i.e., HRS). However, the lack of its adequate conceptualization hampers its concrete operationalization. Autonomy runs deeply in moral philosophy as any other ethical principle (Beauchamp, 2019). Therefore, conceptualize it properly, that is, as an ethical principle, asks to rely on moral philosophy (Bietti, 2020; Giovanola & Tiribelli, 2022a, 2022). The latter indeed helps us to shed light on its multidimensionality and bridge the gap between high-level theory and practical feasibility, namely, to prevent ethics principles from being misunderstood or used as mere labels and therefore becoming trivial, useless, and toothless (Munn, 2022; Rességuier & Rodrigues, 2020).

### The Ethics Principle of Autonomy

As previously mentioned, the current AI ethics principle of autonomy is often vague and opaque. Jobin et al. (2019) provided evidence of this vagueness by analyzing more than 80 ethical frameworks for AI. From this analysis, the ethical principle of autonomy emerges as a core one and it is mainly understood as (i) informational self-determination, (ii) privacy-preserving human control over AI systems, (iii) freedom to withdraw consent, and (iv) freedom from exploitation, manipulation, and surveillance (p.11). Consequently, the most commonly adopted methods of operationalizing it consist of “giving people notice and consent”, “refraining from data collecting and spreading data in the absence of informed consent”, “not reducing options and knowledge of citizens”, “increasing people’s knowledge of AI”, and ensuring “transparency and predictable AI” (p.11). While all these definitions are pertinent, such fragmentation hinders the understanding of what autonomy consists of, what it demands, and how it can be empowered or violated by AI systems. We claim that this opacity is due to the lack of systematic ethical inquiry into the concept of autonomy, which is a notoriously highly complex ethical concept rooted in moral philosophy. To untangle this complexity, in this section, we draw insights on theories on autonomy developed in moral philosophy to unpack the concept of autonomy, highlighting the dimensions it encompasses and the sub-conditions it requires.

In moral philosophy, a prominent notion—also known as the standard conception—of autonomy is that developed within the liberal tradition (Christman & Anderson, 2005). Notwithstanding some conceptual variations, the liberal view on autonomy mainly describes it as rational self-government, control, and independence. From this perspective, autonomy is rooted in the individuals’ capacity to choose and act according to their own interests, reasons, beliefs, and values. More specifically, autonomy is respected and promoted if and only if people can embrace or endorse (i.e., reflectively assess, approve or identify with, or refuse and modify) what guides their choices and, by doing so, self-determine themselves. As follows, the liberal or traditional view on autonomy tends to overlap with self-determination, which in turn is grounded in the individuals’ rational and deliberative capacity to be in control of their actions (or self-govern themselves), insofar as such actions are chosen according to motives that are somehow their own (Frankfurt, 1971; Dworkin, 1988; Ekstrom, 1993; Bratman, 2017; Korsgaard, 2014; Pink, 2016). However, while there is a broad agreement on what autonomy consists of, there is less convergence on what autonomy requires. Within this debate, at least two main conditions are mainly understood as crucial to meeting autonomy: competency conditions and authenticity conditions (Killmister, 2017).

*Competency conditions* refer to a set of cognitive capacities (including psycho-physical abilities) that individuals should own to self-govern or control themselves, which include rational thought to freedom from pathology or systematic self-deception. To foster autonomy, such capacities ought to be respected and/or supported. For example, in the medical field, these conditions are those that enable the individual, once they are provided with certain health information, to be able to act on the basis of that (e.g., information x “smoke damages your health, by affecting a, b, c, etc.” requires competency as the capacity for self-control). In the field of AI, such conditions can be undermined directly when AI decreases such capacities. Some examples in the AI and ethics debate on autonomy’s infringements in this sense include using AI such as RS to enable systematic self-deception (Susser et al., 2019; Natale, 2021), or to steer human behavior according to third-party goals by exploiting cognitive biases or non-rational elements (e.g., health vulnerabilities, traumas, or emotionally-loaded events) (Tiribelli, 2023). Competencies can be violated by AI systems indirectly when there is a human deskilling or loss of capacity due to over-reliance on AI for tasks and decisions or when the information on which one should act is not intelligible to the end-user.

*Authenticity conditions* refer instead to the individuals’ capacity to form authentically or genuinely (i.e., to critically reflect upon and endorse) the reasons, values, interests, preferences, and beliefs motivating their actions. This means that such beliefs, reasons, values, and motivations should not be the product of external manipulative or constraining influences but should be embraced and reflectively endorsed by the subjects as expressing their personal identity. Like competency, authenticity also centers the element of self-reflection, but in a more substantial way.

Competency conditions have been indeed criticized both in moral philosophy as well as in applied ethics, especially in bioethics and medical ethics, as expressing just a formal or procedural account of autonomy. Indeed, they focus solely on the procedure and cognitive means through which a person can come to rationally endorse certain options (i.e., reasons, values, preferences, etc.). Exercising that set of skills is deemed enough to be autonomous. In this sense, they center self-reflection but in a way that is described as procedural independence: they just focus on the procedure while neglecting the importance of questioning whether the options from which a specific person chooses embed what that person (i.e., preferences, values, reasons, etc.) deems truly meaningful. As it has been claimed widely by scholars from feminist ethics (Mackenzie & Stoljar, 2000a, b; Oshana, 2006; Benson, 2005), overlooking the substance in favor of the procedure can lead to easily legitimizing options that embed (historically rooted) values of oppression like unfair and discriminating biases and thereby perpetuating social conditions that are inherently autonomy-undermining as oppressive (i.e., inherently curtail certain options), especially for the most vulnerable and marginalized. Moreover, such conditions fail to account for people who live in constraining health and/or socio-economic conditions, such as those affected by epistemic injustice (Fricker, 2007) and/or physical decline or debilitating pathology but anyway reclaim their autonomy and related dignity (Jaworska, 2009).

This is why competency conditions are not the sole to be considered. The conditions of authenticity express the possibility of the individuals to be autonomous if and only if they can identify with those beliefs, values, preferences, and reasons steering their behavior, endorsing them as motives of their choices and actions (Mackenzie, 2014). In this sense, autonomy is respected and promoted if the options from which a person can choose embed what (value, commitment, project, etc.) that person feels as deeply meaningful (i.e., substantial self-reflection and independence). Looking at the literature on the ethics of AI, such conditions are described to be undermined when AI and RS are used to manipulate individuals (Milano et al., 2020). This is why autonomy is often formalized as “freedom from manipulation and undesired external influences” (Jobin et al., 2019), and is often strictly connected to privacy (Mittelstadt et al., 2016). Such a state of “freedom from interferences” is mainly understood as the protection of individuals’ meaningful space of independence, that is, their informational privacy (Floridi, 2011). Such conditions can also be undermined when AI is used to trigger phenomena such as adaptive preference or belief formation (e.g., RS that pre-select contents or options can lead people to adapt their preferences to match the options available to them) (Adomavicius et al., 2013). Moreover, such conditions can be harmed when the–filtered or recommended—options available are not truly meaningful for the individuals as a result of biased correlations and stereotypes, resulting in a loss of meaningful opportunities.

However, authenticity conditions are also broadly criticized within moral philosophy and ethical theory. Overall, liberal accounts of autonomy as procedural and/or substantial independence are criticized by scholars coming from feminist ethics and other ethical traditions such as communitarianism (Oshana, 2006; Mackenzie & Stoljar, 2000a, b; Westlund, 2009; Gutmann, 1985; Sandel, 2005; Taylor, 1992), as focusing only on an individualistic dimension of autonomy, where the individuals are viewed as independent and self-isolated. Such criticism is particularly strong in an applied ethics field such as medical ethics, in which self-sufficiency and independence sound to be at odds with conditions of assistance, care, support, vulnerability, and dependency that characterize the domains of health and care. Specifically, such scholars emphasize the importance of investigating some further conditions of autonomy in social relations, which they argue are crucial to enable people’s autonomy (in general) and patients’ autonomy (in particular) (Entwistle et al., 2010). Those who advocate for recognizing such relational dimension of autonomy are broadly recognized under the umbrella term of “relational autonomy” (Mackenzie & Stoljar, 2000a, b). Beyond some conceptual differences, the core idea underpinning this view is that authenticity can only occur in social conditions, as it is through the socio-relational (interpersonal) and cultural dimensions that people develop what is meaningful for them and motivates their agency.

Some authors investigated such relational dimension (hereinafter: *relatedness*) of autonomy and found in social recognition and social support some specific **socio-relational conditions** that *ought to* be enabled to respect and promote autonomy (Anderson & Honneth, 2005; Benson, 2005; Westlund, 2009; Govier, 1993). This is because social recognition and support are crucial to motivate one’s own agency, especially when conditions of oppression of various kinds (e.g., related to pathology and aging) can threaten one’s autonomy by undermining one’s own sense of self-confidence, self-trust, and self-respect required for effective agency. Looking at the current literature on AI and RS, such systems can undermine these conditions when RS are designed in ways that lead the individual to self-isolate. If we think about HRS, recommending strict health regimes (e.g., nutritional plans) raises the risk of hampering participation in social and recreational activities. Such conditions can also be undermined when the options available to the subject are filtered or recommended in ways that create homogenization and a lack of socio-cultural diversity (Pariser, 2011). For instance, there are many stereotypical or poorly informed views about older adults, their needs and resources, and their desired way of life (Rubeis et al., 2022). Suppose HRS are designed according to such ageist stereotypes or biases. In that case, HRS can wrongly treat older people as a homogeneous group/category, reducing exposure to socio-relational aged heterogeneity (Fang et al., 2018; Taipale & Hänninen, 2018), hampering the possibility of meeting social support truly felt as meaningful.

Summarizing, through our inquiry into the concept of autonomy, we have shown its multidimensionality (i.e., autonomy is not a black-and-white scenario) and, specifically, the multiple conditions autonomy requires to meet (i.e., protect and promote). In doing so, we have systematically framed the multiple definitions of the principle of autonomy emerging in AI ethics and have clarified that autonomy requires (i) competency, (ii) authenticity, and (iii) relatedness. Additionally, we have shown how each condition can be undermined, clarifying how the ethical challenges raised by HRS can be understood as challenges to the principle of autonomy. Table 2 organizes the sub-conditions required to enable autonomy in relation to the ethical challenges raised by HRS and the key areas of AA to consider in designing HRS. The next section shows how to use this autonomy-based ethical framework for the design of HRS for AA.

**Table 2:** Ethical framework to enable autonomy in the design of HRS for AA

The AI ethics principle	Dimensions	Sub-conditions	Ethical challenges	AA’s aspects in HRS
Autonomy	Procedural independence	Competency conditions	EC1, EC2, EC3 EC4, EC5	PH, CO
	Substantial independence	Authenticity conditions	EC1, EC2, EC3, EC4, EC5	PE
	Relatedness	Socio-relational conditions	EC3, EC6	SO

## From Theory to Practice: Enacting Autonomy in Next-Gen HRS

The previous

section showed that the ethical challenges raised by HRS to individuals’ well-being and AA can be understood as challenges to individuals’ autonomy. In turn, the importance of understanding what autonomy as an ethics principle demands was emphasized, clarifying its key dimensions and sub-conditions, and how they relate to AA’s key aspects. Although HRS are still in their infancy, their applications for general health and medical purposes are growing, along with the sectors of AI-empowered eHealth and telemedicine. Hybrid HRS will soon move from the literature to widespread reality, and ethical analyses and frameworks for their trustworthy development will become pivotal (Etemadi et al., 2022). The protection and promotion of autonomy is not just an extra benefit or an after-the-fact consideration but rather the main goal when designing HRS-based systems to support AA. Therefore, we deem the time is mature to start thinking about next-gen autonomy-enacting HRS. In this section, we briefly propose a few practical strategies to enact autonomy in HRS for AA via the respect and promotion of its key dimensions and sub-conditions.

Respecting and promoting competency conditions (e.g., cognitive and physical capabilities) are crucial when designing HRS to support aging individuals (EC1–EC4). From a practical standpoint (i.e., HRS design), the system must be user-centered and able to understand the user’s conditions and tune its interactions, graphical interfaces, and actual contents accordingly. In particular, functionalities and interactions should be carefully modeled on the “specific” user  (Tiribelli et al., 2023a) and not be excessively generalized, over-simplified (risking to bore the user and cause an early system abandon), over-complicated (instigating rejection and refusal), or over-stimulating, endangering the user psyche. Similarly, the informed consent must be adapted to the user’s capabilities so it can be properly processed and understood.

The respect and promotion of authenticity (i.e., availability of actual meaningful options) are particularly important for users’ well-being during aging. The insurgence of pathologies can hinder individuals’ possibilities to enjoy activities or pursue personal values, beliefs, and personal/social commitments they feel meaningful. To mitigate this issue, HRS might include (i) a space for users to share meaningful information about *how* they want to live their life. Due to the sensitivity of such information and its vulnerability to third-party manipulation, such space should ensure (ii) confidentiality (Tiribelli, 2023), that is, a zone of informational privacy (EC2). This means that what is shared by the users cannot be used for nudging/manipulating them for consumeristic gains. Near-future HRS could elaborate on such meaningful elements, seeking to develop personalized health-centered paths and plans, while avoiding behaviors/side effects harming their meaningful values and preferences (EC1, EC3). When a user’s preferences are at odds with the health goal, HRS should provide users-tailored, intelligible explanations with educational advice to empower the user (EC4). Moreover, such explanations should rely on verifiable mechanisms and sources, leveraging honest/sincere (truth-telling) assumptions, data, and rules. However, sometimes, HRS harbor commercial interests, possibly/inadvertently entailing questionable uses of health-related information. In such cases, as they operate in the health domain, HRS should be subject to duties of transparency and disclosure. Recommendations might also be vulnerable to bias (EC5), and, especially when based on macro-generalizing correlations, they can end up being tailored on stereotypes that neglect formalized categories’ inner heterogeneity. On the one hand, to counter such risk, HRS should include sufficiently articulated user profiling and proper variability of the short-term plans, which are functional to the achievement of long-term objectives. On the other hand, HRS should avoid over-constraining—risking finding no solutions. Therefore, reaching a granularity balance between specification and generalization is imperative.

Ensuring relatedness conditions is pivotal to meeting autonomy, especially for aging individuals, who tend to self-isolate due to the insurgence of pathologies and debilitation. Despite the positive aspects discussed above, connecting people in similar conditions might also lead to epistemic bubbles and exacerbate negative feelings, potentially leading to more isolation and social impoverishment (Nguyen, 2020). Moreover, support offered through health technologies often involves affiliates, caregivers, or HCP and might imply forms of health surveillance or external control, mainly perceived as social pressure. To mitigate such risks, HRS might include mechanisms allowing the users to (i) select their most meaningful inner circles, (ii) provide the possibility to opt out of a given social environment/situation, and (iii) provide feedback, reports, and asking for psychological/social support.

The HRS applications can be considerably heterogeneous and far aside. Therefore, the considerations highlighted above do not presume to be exhaustive. Nevertheless, applying an autonomy-based framework to specific HRS (e.g., for medical purposes) would enable further tailoring (i.e., cognitive-dependent) of the given strategies to increase their context sensitivity and thus expected efficacy.

## Conclusions

This study has investigated the potential of HRS to support AA and their exposure to ethical threats. By analyzing the off-the-shelf technical literature on HRS, we have highlighted that HRS can promote AA if they are functional in four AA’s aspects (i.e., Physical, Personal, Social, and Cognitive). Moreover, we have shown that near-future PT-based HRS deploy a series of techniques. On the one hand, PT-based HRS enable the prevention and personalization of healthcare. On the other hand, they can raise a series of ethical challenges and risks undermining users’ well-being and AA’s aspects (spanning over users’ privacy and freedom of choice to HRS’ accuracy, fairness, and opacity). Elaborating on that, we have suggested addressing such challenges through the AI ethics principle of autonomy (a fundamental tenet of AA, AI, and RS in health). We have then pursued an ethical investigation into the concept of autonomy and showed how it requires conditions of (i) competency, (ii) authenticity, and (iii) relatedness. We have argued how the risks highlighted might hamper such conditions—henceforth, users’ autonomy in aging. By unpacking such an ethical principle, we have proposed an ethical taxonomy or framework of autonomy — filling a current gap in the AI and ethics literature. Finally, we have shown how to operationalize such a framework (autonomy’s dimensions and sub-conditions) by proposing possible practical strategies to design next-gen autonomy-enabling HRS for AA.

Notwithstanding, the HRS currently in use are still in their early stages, characterized by a limited usage of ML and DL. This is particularly the case within the European Union, where regulations for health tech are stricter. However, HRS tend to evolve rapidly. We believe ethically steering HRS’ design to harness their opportunity while counteracting their risks, especially for users’ autonomy in aging, is impelling. Therefore, developing robust ethics for HRS is time-critical, and we hope that the proposed autonomy-based ethical framework helps to pave the way for the upcoming efforts.

## Abbreviations

HRS: Health recommender systems
ML: Machine learning
AA: Active aging
DL: Deep-learning
AI: Artificial intelligence
CF: Collaborative filtering
PH: Physical
CB: Content-based filtering
PE: Personal
HR: Hybrid recommendation
PT: Persuasion technologies
KB: Knowledge-based recommendation
RS: Recommender systems
EC: Ethical challenges
SO: Sociale
CO: Cognitive
WHO: World Health Organization
HCP: Healthcare providers

## Footnotes

1 It is important to highlight that autonomy is an ethical principle that tends to be prominent in Western cultures more than in other parts of the world. However, recent contributions have highlighted the centrality of autonomy, and especially of relational autonomy (see Sect. 3.2), also in non-Western perspectives (e.g., Ubuntu Ethics), showing the importance of analyses that consider it beyond its mainstream Western understanding as individual self-determination and independence for a truly inclusive and ethical design of AI-based systems (Mhlambi & Tiribelli, 2023).
